# Exploitation of Ubiquitous Wi-Fi Devices as Building Blocks for Improvised Motion Detection Systems

**DOI:** 10.3390/s16030307

**Published:** 2016-02-27

**Authors:** Francesco Soldovieri, Gianluca Gennarelli

**Affiliations:** Institute for Electromagnetic Sensing of the Environment, National Research Council, Via Diocleziano 328, Napoli 80124, Italy; soldovieri.f@irea.cnr.it

**Keywords:** improvised motion detection system, wireless signals

## Abstract

This article deals with a feasibility study on the detection of human movements in indoor scenarios based on radio signal strength variations. The sensing principle exploits the fact that the human body interacts with wireless signals, introducing variations of the radiowave fields due to shadowing and multipath phenomena. As a result, human motion can be inferred from fluctuations of radiowave power collected by a receiving terminal. In this paper, we investigate the potentialities of widely available wireless communication devices in order to develop an improvised motion detection system (IMDS). Experimental tests are performed in an indoor environment by using a smartphone as a Wi-Fi access point and a laptop with dedicated software as a receiver. Simple detection strategies tailored for real-time operation are implemented to process the received signal strength measurements. The achieved results confirm the potentialities of the simple system here proposed to reliably detect human motion in operational conditions.

## 1. Introduction

In recent years, wireless technology has become very pervasive by covering different application fields, ranging from communications to surveillance, security, medicine, and environmental monitoring. Significant efforts have been carried out to address the challenging problems of detection, localization, and tracking of targets in complex scenarios, such as urban areas or indoor environments. For instance, active radar systems have been designed and enhanced by specific data processing algorithms for urban sensing [1,2,3], through-wall imaging and tracking [4,5,6,7], building interior imaging [8,9], concealed weapon detection [10,11], detection of buried ordnances [12,13], and infrastructure monitoring [14,15].

The intrinsic difficulties related to the localization and tracking of targets in a complex electromagnetic scenario for security and military applications have motivated the development of sophisticated and expensive radars; as a consequence these devices are not suitable for civilian applications where a large scale and long-term sensor deployment is usually demanded.

In this framework, the rapid and enormous diffusion of mobile computing and wireless devices (e.g., Wi-Fi cards, smartphones, *etc.*) and sensor networks, has recently triggered pioneering research studies with the goal to replace ad-hoc sensors for remote sensing applications with cheaper wireless devices not specifically designed for remote sensing purposes [16,17,18,19]. These sensors have low cost, narrow bandwidth, and power consumption constraints. Moreover, they are typically only capable to measure the received signal strength (RSS) of the wireless signal.

In a different, but related, context passive radar systems are also attracting a growing interest owing to their low cost, covertness, anti-jam features, and effective exploitation of opportunistic sources in both heavy and scarce population areas [20,21,22,23]. In urban areas, these opportunistic sources can be local to the target surroundings, such as Wi-Fi or Wi-Max, or non-specific serving wide areas, such as digital audio broadcasting, cell-phone base stations, and satellite-borne illumination.

Similar trends have also arisen in healthcare applications, where active radar systems have been developed for remote patient monitoring and elderly care. Actually, these devices allow monitoring vital parameters (breathing and heartbeat) of human subjects and living animals [24,25,26,27]. Furthermore, radar signatures can be profitably exploited to recognize human gait [28,29] and monitor the movements of elderly subjects in smart homes with the aim to detect falls [30,31]. Despite the current technological solutions being very promising, in most cases the available systems are designed for specific applications and their costs is still prohibitive to permit a large scale diffusion.

In view of previous considerations, a growing attention has been recently devoted to the assessment and potential exploitation of ubiquitous wireless devices (e.g., smartphone, tablets, *etc.*) in different applicative contexts, e.g., see [32,33,34,35,36] for representative examples. In particular, a device-free passive localization architecture has been proposed in [32] in order to detect, localize, and track a human subject in an indoor environment. A human detection method based on RSS variations has been developed and incorporated into an existing platform for residential smart energy management [33]. A Wi-Fi vision system based on compact devices with narrow bandwidth, low power, and accessible to non-military entities has been presented in [34] in order to detect targets behind building walls. An accurate indoor positioning system for commodity mobile devices, with no specialized infrastructure or fingerprinting has been reported in [35]. A respiratory monitoring system consisting in ubiquitous off-the-shelf Wi-Fi-enabled devices has been developed in [36] for breathing rate estimation, as well as apnea detection. Moreover, recently, there has been a growing attention in the area of Wi-Fi-based device-free human motion recognition using channel state information (CSI) and raw samples in place of RSS (e.g., see [37,38,39]).

The present paper moves in the above sketched scenario and is concerned with a feasibility study on the usage of ubiquitous Wi-Fi devices to detect human motion in indoor areas. Therefore, it is relevant to medical mobile sensing, where the attention is focused on the use of smartphones and other connected devices for remote sensing in medicine and elderly care applications. In addition, the proposed technological solution is valuable for the detection of movements as indicator of human presence in inaccessible environments after crisis events (e.g., buildings on fire), or in intrusion detection for security purposes.

Nowadays, several types of sensors are available on the market to detect human presence. Popular examples are passive infrared sensors, which detect the heat radiated by human body, or radar-based devices that detect the presence of a person based on the scattered RF signals. These kinds of devices are very reliable but usually require the installation of *ad hoc* components into the surveillance region.

In this work, we exploit widely available Wi-Fi devices, specifically a commercial smartphone as the Access Point (AP) and a laptop with dedicated software as the Receiving Terminal (RT), to enable the improvised detection of moving people. The monitoring system at hand is very simple and costless as it reuses general purpose devices already available in public or private areas. From this point on, this system will be named as Improvised Motion Detection System (IMDS) to stress the fact that it is composed by opportunistic devices instead of dedicated hardware. For such IMDS, RSS data are recorded over time and human presence is inferred by taking advantage of the changes in radiowave signal levels caused by shadowing and multipath phenomena due to human motion. Experimental tests have been carried out in an indoor environment and the collected data have been processed by two different data processing algorithms. Regardless of the processing strategy, it is shown that this IMDS is capable to reliably detect the movement of people inside the surveillance region.

The paper is organized as follows. In Section 2, the physical principle at the basis of the motion sensing process is reviewed and supported by analytical/numerical electromagnetic scattering models. The measurement system is described in Section 3, while the data processing approaches and experimental results are reported in Section 4. Concluding remarks and perspectives follow in Section 5.

## 2. Electromagnetic Scattering Models

**Figure 1:**
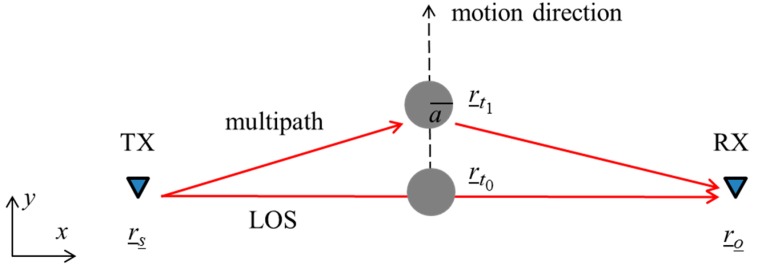
Two-dimensional free-space scenario showing the scattering phenomena arising when a metallic target moves between a transmitting and a receiving wireless device. Red arrows denote two ray propagation paths from TX to RX.

The working principle at the basis of the motion detection process exploits electromagnetic scattering, which models the interaction between the electromagnetic signals emitted by the wireless devices and the targets (e.g., people) in the region under surveillance. An exemplification of this principle is reported in the following with regard to the simplified two-dimensional (2D) geometry depicted in Figure 1. The scene consists of a transmitter (TX) and a receiver (RX) located in free-space at positions ${\underline{r}}_{s}$ and ${\underline{r}}_{o}$, respectively. In view of the narrow-band nature of common devices, both TX and RX are supposed to operate at a single frequency ω. The TX is modeled as a filamentary electric current directed along the *z*-axis. A metallic cylindrical target with radius *a* moves from the point ${\underline{r}}_{t0}$ to ${\underline{r}}_{t1}$ along the direction perpendicular to the line passing between TX and RX (*y*-axis). It must be underlined that the geometry in Figure 1 emulates a human subject that moves in the region between the transmitter and receiver. The model is very simplified because the human body cross-section is assumed invariant along the *z*-axis and the effect of real geometrical and electromagnetic properties of the human body are neglected. Despite this simplification, this simple model provides good physical insight into the motion sensing process.

The transmitter radiates an incident field $E^{i}$ that impinges on the target, thus inducing electric currents over its surface. These induced currents radiate a scattered (secondary) electric field $E^{s}$, which depends on the electromagnetic properties of the target and on its relative position with respect to the transmitting and receiving antenna. The total field $E^{t}$ at the receiver is given by the coherent sum of the incident field (*i.e.*, the field in absence of any target) and the scattered field, * i.e.*:

(1) $$E^{t}=E^{i}+E^{s}$$

According to [40], the quantities involved in Equation (1) have the following closed-form expressions:

(2) $$E^{i}\left({\underline{r}}_{o}\right)=-\frac{\beta^{2}I}{4\omega \varepsilon }H_{0}^{\left(2\right)}\left(\beta \left|{\underline{r}}_{o}-{\underline{r}}_{s}\right|\right)$$

(3) $$E^{s}\left({\underline{r}}_{o}\right)=\frac{\beta^{2}I}{4\omega \varepsilon }\overset{n=\infty }{\underset{n=-\infty }{\sum }}H_{n}^{\left(2\right)}\left(\beta \left|{\underline{r}}_{t}-{\underline{r}}_{s}\right|\right)\frac{J_{n}^{\left(2\right)}\left(\beta a\right)}{H_{n}^{\left(2\right)}\left(\beta a\right)}H_{n}^{\left(2\right)}\left(\beta \left|{\underline{r}}_{o}-{\underline{r}}_{t}\right|\right)e^{jn(\phi -\phi ')}$$

where $\beta =\frac{\omega }{c}$ is the propagation constant in free-space (*c* is the speed of light), ε is the free-space permittivity, $H_{n}^{\left(2\right)}$ and $J_{n}^{\left(2\right)}$ are the Hankel and Bessel functions of second kind and order *n*, and ${\underline{r}}_{t}$ is the target position along its path. Moreover, ϕ' and ϕ represent the directions under which the TX and RX are seen by the target [40].

The electromagnetic power detected by the receiver is proportional to the square amplitude of the total electric field (In practice, the detected power depends on a number of factors among which the antenna patterns of the TX and RX, as well as the RX input circuit. For sake of simplicity, these effects are neglected in this study), *i.e.*,

(4) $$P^{t}=10log_{10}|E^{t}{|}^{2}$$

The RF signal power defined by Equation (4) exhibits an oscillating behavior as the target moves between the source and receiver, depending on how the incident and scattered fields combine at the receiver (constructively or destructively). To visualize this phenomenon, we report a numerical example in the following based on Equations (1)–(4). With regard to the geometry in Figure 1, we consider a TX and RX operating at 2.45 GHz, which are located along the *x*-axis at distance of 3.5 m one from each other. A metallic cylinder having radius of 0.2 m and placed at the mid-point between the TX and RX moves along the *y*-axis covering a distance of 1.5 m.

**Figure 2:**
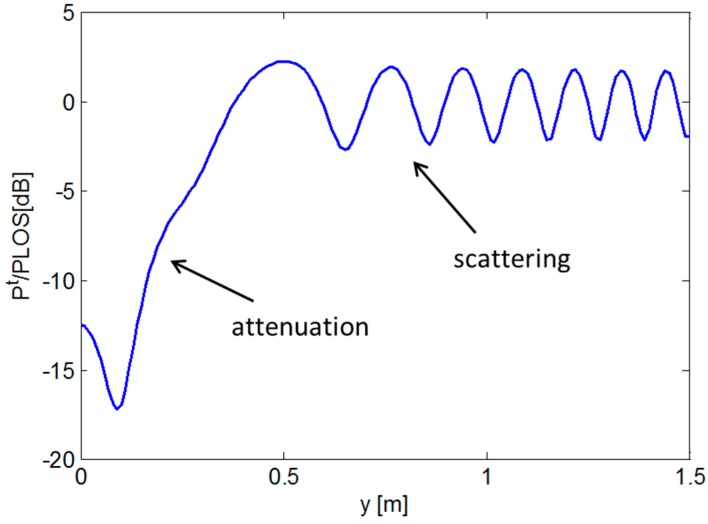
Ratio between total RF power and LOS power *versus* distance covered by the target. 2D free-space scenario.

The behavior of RF signal power is illustrated in Figure 2. Note that the total field power has been normalized to the power in Line of Sight (LOS) conditions, *i.e.*, in absence of target, in order to better highlight the effect of the target’s movement. As can be observed, when the target is close to the *x*-axis, the RF signal undergoes a significant attenuation because the LOS path is blocked (see Figure 2). On the other hand, when the target moves away from the *x*-axis, the signal level increases since the LOS path is restored. Note that the 0 dB value corresponds to the RF LOS power and the oscillations of few dBs around such a value depend on the way incident and scattered waves interfere at the receiver (see Figure 2).

**Figure 3:**
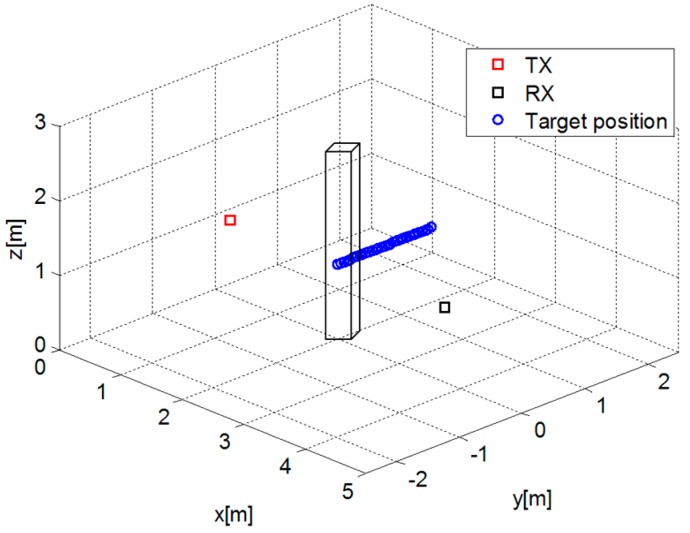
Geometry of the 3D free-space scenario.

A similar analysis has been performed also in the case of a 3D free-space scenario by means of a numerical full-wave simulator implementing the Finite-Difference Time-Domain (FDTD) method [41]. The scenario depicted in Figure 3 features a TX located at (0, 0, 1) m and a RX placed at (3.5, 0, 1) m corresponding to a distance between them equal to 3.5 m. Both TX and RX antennas operate at 2.45 GHz and are modeled as Hertzian dipoles directed along the *z*-axis to achieve the best polarization match. A metallic parallelepiped with size 0.2 m × 0.4 m × 1.80 m (simulating a human subject) moves along the *y*-axis between the source and receiver covering a distance equal to 1.5 m.

**Figure 4:**
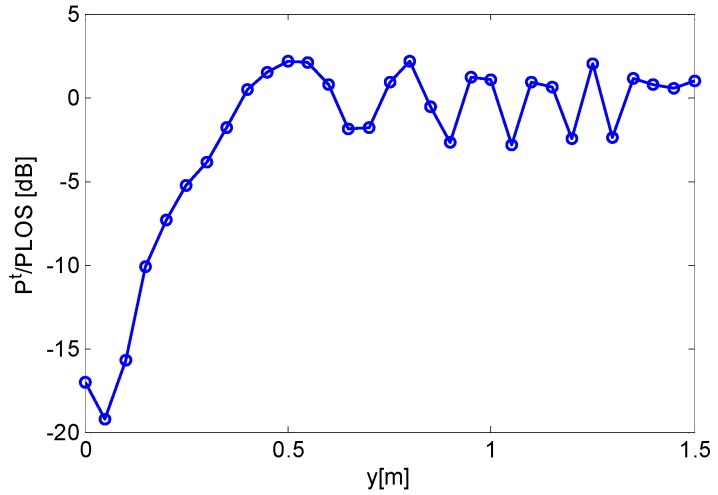
Ratio between total RF power and LOS power *versus* distance covered by the target. 3D free-space scenario.

The ratio between the total RF power and LOS power is depicted in Figure 4. It can be observed that the attenuation and scattering phenomena are very similar to the ones evaluated for the 2D scenario.

The principle at the basis of the motion detection strategy is the exploitation of the variation of the RF signal power due to the attenuation and scattering phenomena arising when the target interacts with wireless signal. These variations are in practice observed in the RSS measurements since this datum is usually made available by commercial wireless devices.

It is worth noting that that the RSS is a quantity expressed in dBm, which provides an indication of the power level received by a wireless device. However, according to the IEEE 802.11 standard [42], there is no unique relationship between RSS and signal power in dBm; consequently, RSS data provided by different manufacturers are not directly comparable [43]. This fact is one of the main limitations to the integration of different types of ubiquitous wireless devices (possibly from different manufacturers) into a system for target detection and localization [43].

## 3. IMDS Description

In this section, we provide a description of the measurement setup employed for the experimental tests. The Microsoft Lumia 535 smartphone set in hotspot modality has been used as the AP operating in the Wi-Fi band according to IEEE standard 802.11 b/g/n. A Lenovo laptop equipped with the wireless adapter Intel-N 7260 has been employed as the RT to collect the RSS data. The freeware Wi-Fi monitoring software Homedale [44] has been used to visualize and record the RSS measurements to be given as input to the motion detection algorithm. This software provides RSS information for the wireless link of interest with 1 dB resolution and a sampling time defined by the user. The sampling time has been set equal to 2 s for the experiments described in this work.

**Figure 5:**
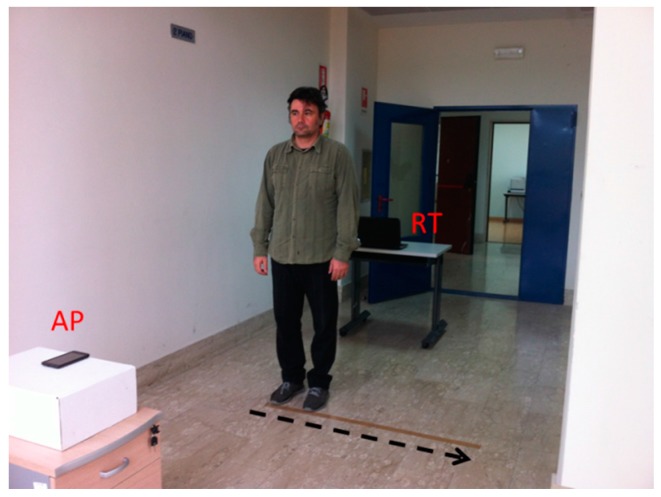
Photo of the experimental set-up. The arrow indicates the movement direction.

A preliminary measurement campaign has been executed in a relatively quiet indoor environment (see Figure 5) in order to compare the theoretical and real RF signal strengths when a human stands in the region between the AP and RT. The two devices (AP and RT) have been positioned at a height of 0.8 m over the floor and facing each other at a distance of 3.5 m, similarly to the numerical model presented in Section 2 (see Figure 3). The subject position has been varied along the direction perpendicular to the LOS between AP and RT following a path 1.5 m long with a spatial step of 0.1 m (16 measurement points), where the first position corresponded to the person blocking the LOS. For each subject position, RSS data have been collected over a temporal window of 60 s with a step of 2 s. After, the data have been averaged over time to have a reliable representative value of the RF signal strength. In order to compare experimental data with theoretical values, the RSS measurements in presence of the subject have been normalized to the RSS data collected in LOS scenario (without subject).

**Figure 6:**
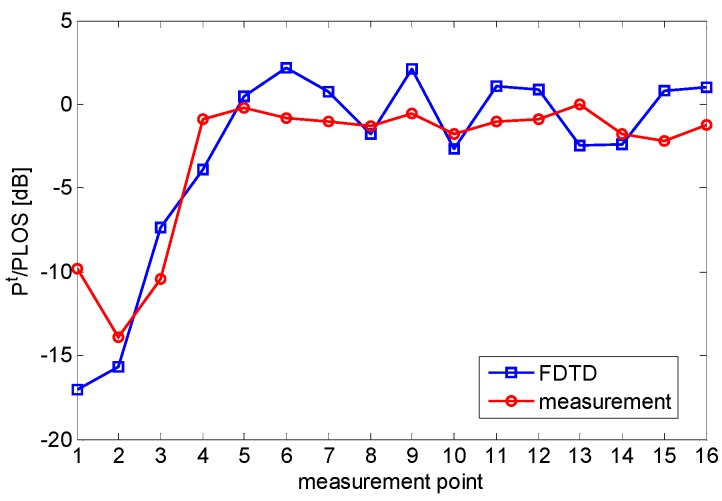
Comparison between simulated and measured ratios of total RF signal power and LOS power.

The results plotted in Figure 6 highlight similar trends for numerical simulation and experimental data. In detail, the RF signal attenuation phenomenon is clearly observable in the first five measurement points after which the RF power becomes oscillating as the subject moves away from the LOS between the AP and RT.

Note that the differences in the amplitude of RF signal oscillations are ascribable to the poor quantization (1 dBm) of RSS measurements and to the fact that the FDTD model is idealistic since it does not account for AP and RT antenna patterns as well as the true geometrical and electromagnetic characteristics of the scenario and of the human subject. In this sense, the comparison among the curves plotted in Figure 6 should be seen as a qualitative one. Nevertheless, it is very insightful since it provides experimental evidence of the irregularities in the radiowave pattern due to shadowing and multipath.

## 4. Data Processing Approach and Results

**Figure 7:**
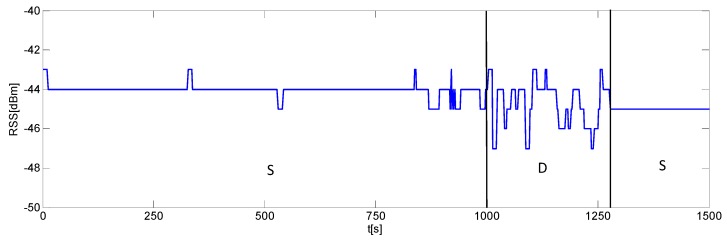
Example of a RSS record in a time varying indoor environment. The scene is static (S) in the interval [0, 1000] s, dynamic (D) in the interval [1000, 1280] s, and again static (S) in the interval [1280, 1500] s.

Experimental tests assessing the effectiveness of the IMDS have been carried out in the indoor area shown in Figure 5. Several datasets have been recorded with the human subject moving and standing still during the signal acquisition. Figure 7 is an example showing the RSS temporal characteristics over a time window of 1500 s. Specifically, the subject was stationary in the interval [0, 1000] s, then he moved in the window [1000, 1280] s, and finally he was again stationary in the interval [1280, 1500] s. As can be observed, the RSS fluctuations are reduced when the scene is stationary. Conversely, the RSS exhibits a stronger variability as the human subject walks. This outcome is consistent with previous analysis and in agreement with the results of recent literature on the topic (e.g., see [35,45]).

The goal of the motion detection algorithm is to exploit the time varying features of RSS data, which are quantified by a suitable metric based on a short-time (local) analysis of the signal. The metric adopted in this work is the standard deviation of the RSS time series. Specifically, the algorithm tracks the standard deviation between the first and last measurement over a moving time window and human motion is detected if the standard deviation is larger than a predefined threshold.

Let *y_n_ n = 1,...,N* be the whole RSS data sequence with *N* being the total number of samples. Denote with *w_n_*, *n = 1,...,N_w_*, a windowed version of the signal *y_n_*, with *N_w_* being the number of samples in the window. Such a window is progressively translated forward by one sample up to the last measurement in the RSS time-series. The motion detection algorithm consists in the following two steps:

1. Computation of the standard deviation for each time window:
  (5) $$\sigma_{m}=\sqrt{\frac{1}{N_{w}}\overset{N_{w}}{\underset{n=1}{\sum }}{\left(w_{n}-{\bar{w}}_{m}\right)}^{2}}\;m=1,\ldots N-N_{w}+1$$
  where ${\bar{w}}_{m}$ is the average value of the sequence *w_n_* over the current window *m*; and
2. Movement detection if the standard deviation is greater than a threshold , *i.e.*:
  (6) $$Y_{m}=\{\begin{array}{c} 1\;\;\;\;\sigma_{m}\geq \gamma \\ 0\;\;\;\;\sigma_{m}<\gamma \end{array}\;m=1,\ldots N-N_{w}+1$$
  where $Y_{m}\in \{0,1\}$ is the binary detector output.

In this study, we have considered a moving window having size *N_w_* = 8 and *γ* was set to 0.8 after analyzing several datasets with no human moving in the scene. Of course, the threshold value depends on the measurement configuration as well as the layout of the scene, thus it needs to be recalculated every time that measurement conditions change.

We compare the detection results achieved by applying the rule in Equation (6) with the approach proposed in [45] based on histogram analysis of instantaneous RSS variation, *i.e.*, $\Delta y_{n}=y_{n+1}-y_{n}$. The method exploits the fact that the histogram becomes more spread in a dynamic environment, thus motion can be detected by exploiting the differences between histogram features.

**Figure 8:**
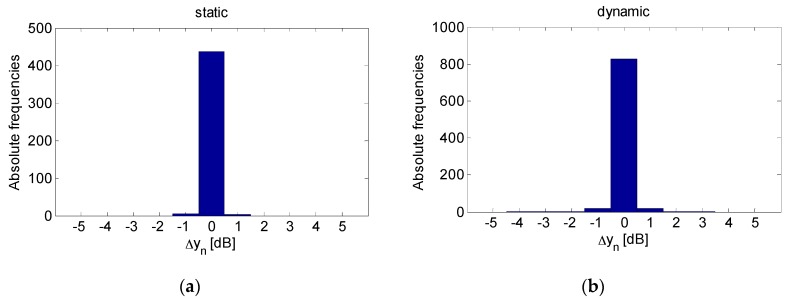
Histogram of instantaneous RSS variation for (**a**) a static scenario and (**b**) a dynamic scenario.

For instance, the histograms depicted in Figure 8 corresponding to data displayed in Figure 7, highlight that values fall within the interval [−1, 1] when the scenario is static (left plot). Conversely, in a dynamic environment, there are several fluctuations outside the range [−1, 1]. According to [45], a simple strategy for motion sensing consists in counting the number of fluctuations falling in [−1, 1] over a moving window of $N_{w}$ samples. For each window, the algorithm decides that there is motion if the percentage of such fluctuations falls below a threshold α. Based on experimental analysis of data collected in a static environment, the threshold α was set equal to 0.95.

**Figure 9:**
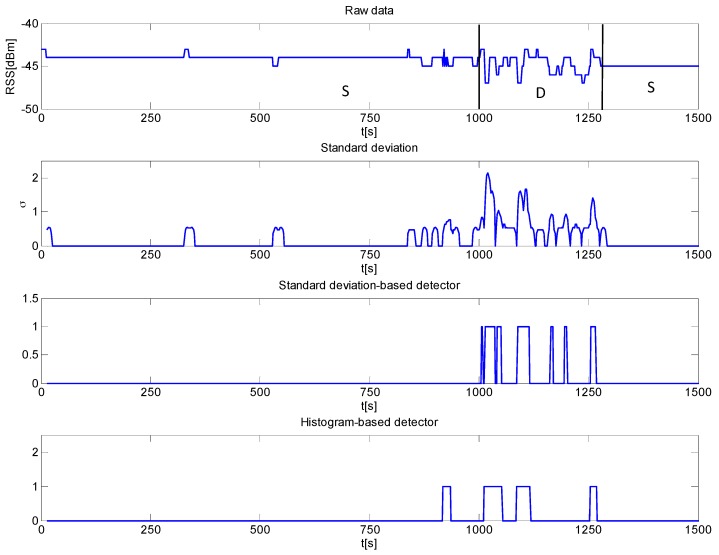
Measurement results and detector outputs for the scene with a subject moving in the interval [1000, 1250] s.

We processed the dataset of Figure 7 relevant to a subject moving in the interval [1000, 1250] s by applying both motion sensing algorithms. The achieved results are plotted in Figure 9. The first panel displays the data, while the second panel represents the behavior of the local standard deviation of RSS. As expected, the standard deviation exhibits several peaks in the time interval where the subject is moving, while it is mostly zero when the scene is static. The outputs of the standard deviation-based detection rule are reported in the third panel of Figure 9. It can be noticed that motion is reliably detected because the output is always zero save for the time interval [1000, 1280] s where it is frequently equal to one. For comparison purposes, the fourth panel of Figure 9 shows the output of the detection algorithm based on histogram analysis. In this case, the results are comparable to those achieved with the standard deviation–based detector, with the exception of a false alarm generated around *t* = 900 s. The false positive rate is found to be 0 with standard deviation detector and 0.007 with histogram-based detector. As regards the false negative rates, they are equal to 0.57 for standard deviation detector and to 0.63 for histogram-based detector. Note that the reason why false negative rates are not low is only due to the fact that, as shown by Figure 6, the system is mostly sensitive to human movements obstructing the LOS. As the human subject moves away from LOS, e.g., at a distance greater than 50 cm, the variation of RSS is below the resolution of the adopted measurement system. The above mentioned false negative rates do not account for this fact; hence, they are significantly overestimated.

**Figure 10:**
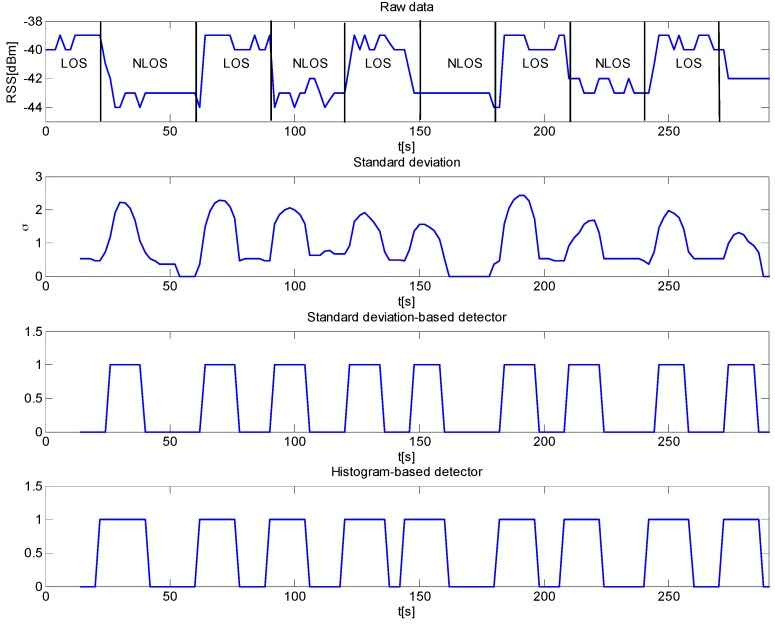
Measurement results and detector outputs for the scene with a subject periodically interrupting the LOS between AP and RT.

The results of another representative experimental test are reported in Figure 10. In this case, the subject periodically stopped between the AP and RT interrupting the LOS for about 30 s. This shadowing phenomenon is clearly observable and highlighted by NLOS intervals in RSS data (first panel of Figure 10). Consistently, the standard deviation locally peaks in correspondence of the transitions from a LOS to a NLOS condition and viceversa. As for detection results, both algorithms yield the same results, thus confirming the reliability of the IMDS.

Finally, it is worth noting that the system has been found capable to detect human movements for distances between the AP and RT up to 8 m in a free room. However, this working range is variable and dependent on the geometrical and electromagnetic characteristics of the environment and on the AP transmit power.

## 5. Conclusions

This work has demonstrated the potential usage of widely available Wi-Fi devices as valuable tools to develop improvised motion detection systems. This concept is of significant interest in many applicative fields such as security, surveillance, medical mobile sensing, and elderly care. By exploiting the variations of the radiofrequency signal power caused by shadowing and multipath effects and simple data processing algorithms, the system has been shown to provide reliable indications about human movements. The system is very simple and costless and it is capable of real-time operation due to the inherent simplicity of the considered data processing.

It must be stressed that the system is capable to collect RSS data with a quantization step of 1 dB. Therefore, it is mainly sensitive to human movements that obstruct the line of sight (LOS) between the access point and the receiving terminal. As the subject moves away from LOS, e.g. at a distance higher than 50 cm, the variation of received power due scattering effects is small and below the resolution of the measurement system. We have found that the system can detect movements of a walking human on the order of few ten centimeters. It is envisaged that this result may be improved by using more accurate hardware at the receiving terminal. 

As for the working range, the system is capable to detect human subjects walking in a free room for distances between the access point and the receiver up to 8 m. Of course, the working range depends on the geometrical and electromagnetic characteristics of the environment and on the access point transmit power.

Future research developments regard two main directions. The first one is concerned with a more focused activity regarding specific applications in elderly care and medical mobile sensing, with a deep survey of the most suitable hardware and data processing.

The second one is concerned with the development of suitable approaches tackling the problem of more pronounced variability of the Wi-Fi signal when nearby devices operate in the same radio channel since this signal variability can be very detrimental for the movement detection and localization [43].
